# Considerations of HLA, Renal Failure, Valproic Acid Use, and Current Treatment Guidelines in Clozapine-Induced Agranulocytosis

**DOI:** 10.1155/2021/8816390

**Published:** 2021-02-20

**Authors:** Melissa Sussman, Michael Epifania, Derrick Eng, Yun Jae Cho, Richard Steward

**Affiliations:** ^1^Lake Erie College of Osteopathic Medicine, 1858 W Grandview Blvd, Erie, PA 16509, USA; ^2^St. John's Episcopal Hospital, Department of Family Medicine, Queens 327 Beach 19th St., NY 11691, USA

## Abstract

Clozapine, the choice atypical antipsychotic for refractory schizophrenia, schizoaffective disorder, and bipolar disorder, has been shown to reduce positive and negative symptoms of schizophrenia. Clozapine, though beneficial in reducing the need for hospitalization, rehabilitation, and health care costs, is known as a drug of last resort due to its potential adverse event of clozapine-induced agranulocytosis, which holds a case fatality rate between 4.2 and 16%. Herein, we describe a female patient with longstanding schizoaffective disorder and chronic kidney disease who suffered from clozapine-induced agranulocytosis after failing two other atypical antipsychotics. Retrospective considerations of this case and management highlight risk factors such as HLA status, renal failure, and concurrent valproic acid use which presently do not have official screening, guidelines, or restrictions in place when prescribing clozapine. Additionally, there are no specific clozapine-induced agranulocytosis management recommendations such as G-CSF/filgrastim dose, timing of bone marrow aspirate and biopsy, and use of concomitant valproate. We propose that further comprehensive official screening, monitoring, and guidelines in the prescribing of clozapine, and further guidelines in the treatment of clozapine induced agranulocytosis, could increase the cost-effectiveness of clozapine treatment, and decrease the incidence, and morbidity of this feared adverse event.

## 1. Introduction

Clozapine, a dibenzodiazepine antipsychotic exhibiting weak dopaminergic activity and atypical pharmacological properties, is the choice antipsychotic for refractory schizophrenia, schizoaffective disorder, and bipolar disorder [1–4]. Advantages of clozapine include decreased positive symptoms of schizophrenia such as voices and hallucinations and decreased negative symptoms such as blunted affect and suicidal behavior [5]. The decreased positive and negative symptoms result in reduced need for hospitalization, rehabilitation, and subsequent health care costs [6]. However, known and feared disadvantages include neutropenia and life-threatening agranulocytosis [7, 8].

A benign form of neutropenia with an absolute neutrophil count (ANC) < 1500 cells/*μ*L [9] is known to occur more frequently than clinically significant, agranulocytosis, where ANC < 500 cells/*μ*L [10]. A high risk of severe and/or opportunistic infections is noted to be associated with ANC <200 [9]. In 2008, clozapine-induced agranulocytosis (CIA) had a known rate of seven cases per one million [11]. In 2006, the case fatality caused by CIA was estimated to be 4.2% to 16% [12]. In 2016, the observed mortality rate ranged from 0.1 and 0.3 per thousand, and the case fatality rate was estimated to be between 2.2 and 4.2 [13]. Standard established protocol includes immediate discontinuation of clozapine should white blood cell count reach <500 ANC [10, 14]. Patients' WBC and ANC must be monitored daily until WBC > 3000/mm^3^ and ANC > 1500/mm^3^ [7].

As more cases of CIA are occurring, more information concerning prescribing this efficacious antipsychotic as well as the treatment of CIA has become available. Here, we describe a patient with longstanding schizoaffective disorder who suffered from CIA after being transitioned to clozapine due to failed trials of risperidone and olanzapine. Retrospective considerations of her placement on clozapine and subsequent management of her CIA brought into question many factors which do not have clear guidelines, including official screening of risk factors which increase likelihood of CIA, as well as clear clinical management for this feared adverse event. This case report provides a spotlight on many factors such as screening for chronic kidney disease (CKD), human leukocyte antigen(HLA) typing, concurrent valproate administration due to potential CYP interactions, and management considerations such as dosing for filgrastim/G-CSF and timing of bone marrow biopsy. This analysis aims for further study and ultimately future implementation of screening and management guidelines for better informed management of refractory schizophrenia treatment, reduced cases of CIA, and improved management of CIA should it occur.

## 2. Case Presentation

A 60-year-old Caucasian female nursing home resident was brought in by EMS to the ED due to disorganized behavior, auditory hallucinations, and disorganized speech with loosening associations and was admitted to the inpatient psychiatric unit. Past psychiatric history included longstanding schizoaffective disorder. As a psychiatric inpatient, after having failed treatment with risperidone and olanzapine, the patient was started on clozapine 25 mg PO daily and was titrated to 150 mg PO BID over the course of 28 days. Recorded WBC on day 28 of treatment was 4.5 × 10^9^/L (4.5 to 11.0 × 10^9^/L). On the 35^th^ day of clozapine treatment, the patient reported sore throat, nausea, diarrhea, and difficulty falling asleep. WBC was found to be 0.2 × 10^9^/L (4.5 to 11.0 × 10^9^/L), and ANC was 0.05 [1.5 to 8.0 (1,500 to 8,000/mm^3^)]. The valproate level was 36.5 (50-100). Subsequently, the patient was transferred to the inpatient medicine floor this same day due to clozapine-induced leukocytopenia (day of transfer to medicine/Treatment day 0).

Past medical history included arthritis, deep vein thrombosis, chronic kidney disease (CKD) (stage 3), right bundle branch block, left anterior fascicular block, syncope, and epilepsy. On admission, patient was alert and oriented ×2 to self and place. The patient reported sore throat, nausea, diarrhea, and difficulty falling asleep. The patient denied headache, shortness of breath, abdominal pain, dysuria, or urinary frequency. The patient's allergies included penicillamine, penicillin, and shellfish. Active medications included sodium valproate, amlodipine, docusate, and polyethylene glycol. Physical exam was unremarkable, and vitals were within normal limits (See Table 1).

### 2.1. Treatment Course

On the day of transfer to medicine (Treatment day 0-Table 1), clozapine was immediately discontinued, the patient was placed in reverse isolation, and a throat culture was taken. On Treatment day 1, upon recommendation from hematology, the patient received filgrastim/G-CSF 300 mcg subcutaneously. The patient continued this dosage of filgrastim for two days along with home dose of valproate.

On Treatment day 3, the patient spiked a fever of 103.1°F. A fever source was investigated, and an uncomplicated cellulitis on the abdominal wall was found. Filgrastim was adjusted to a dose of 800 mcg subcutaneous daily (see Table 1), and the patient was administered acetaminophen, aztreonam, clindamycin, and gentamicin while monitoring the kidney function.

Gentamicin was discontinued on Treatment day 5 (two days after initiation) when eGFR was 17.24 indicative of AKI stage 4 (see Table 1). Aztreonam and clindamycin were continued till Treatment day 6, and micafungin and meropenem administration began on Treatment day 7.

Patient status stabilized on Treatment day 10 when her ANC count rose to 1.8, up from 0.4 on Treatment day 9 (See Table 1). The patient remained hospitalized an additional two days to correct electrolyte abnormalities.

## 3. Discussion

As CIA has become more prevalent, it has been noted that risk factors and poor prognostic indicators include female sex, increasing age, ANC < 100, preexisting renal, cardiac or inflammatory disease comorbidities, and sepsis. [14]. Considering these factors, the 60 years of age in our female patient, her CKD, the acquisition of cellulitis during her treatment, and ANC of 5, we were satisfied with the treatment course and outcome. However, other factors have been noted in literature to potentially increase susceptibility of CIA. Specific human leukocyte antigen (HLA) has been identified for increased susceptibility to CIA. Potential CYP reactions in valproate, which remain unclear in literature, could also potentially increase risk of CIA. And certain treatment guidelines of CIA remain vague in literature. Clinical screening either separately or combined for many of these risk factors should be established. Additionally, consideration for a standardized protocol for timeline and dosing of treatment for CIA should be established.

### 3.1. Screening: Renal Failure and HLA

Though CIA is an adverse event which occurs in less than 1% of adults with psychiatric conditions [15], more research is being conducted to determine risk factors which may increase the likelihood of patients developing this condition. By screening for risk factors, we may mitigate the occurrence of these events. It is noted that our patient who had a past medical history of CKD was dosed with gentamicin upon initial fever of 103.1°F on Treatment day 3. Though gentamicin has known nephrotoxic effects and was thus discontinued to prevent further kidney toxicity, many case reports have suggested that clozapine may induce and/or exacerbate renal failure and nephritis [16–19]. While further research is necessary to quantify the precise risk of clozapine on the kidney function, prior to initiating a trial dose of clozapine, including a thorough history, measuring a patient's kidney function, or eGFR, and BUN/creatinine panel in addition to weekly monitoring of clozapine and ANC levels may reduce exacerbation of CKD.

Associations between susceptibility of CIA and human leukocyte antigen (HLA) were initially reported in the 1990s [20]. In 2014, HLA-DQB1 and HLA-B alleles were specifically implicated in amino acid changes responsible for patient's susceptibility to CIA [21]. Currently, there is no guidance on mandatory panels or screening for HLA alleles prior to administration of clozapine though such screening has been shown to be health and cost-effective [15]. While the past medical history indicated that our subject had been diagnosed with arthritis, we did not investigate which type of arthritis. We performed HLA screening towards the end of her CIA treatment and discovered that our patient was, indeed, HLA-DQB1 and HLA-DQ2 positive. Had we implemented HLA screening prior to initiating clozapine, we could have potentially avoided her CIA.

### 3.2. Management Protocol for Clozapine-Induced Agranulocytosis

While standard procedures for the management of CIA have been established, many of the details remain either up to physician determination or unestablished guidelines. Filgrastim/G-CSF has been shown to decrease the recovery time associated with agranulocytosis [7]. However, there is not an established dose for filgrastim for the treatment of CIA. There are established guidelines for filgrastim/G-CSF use in patients suffering from a number of immunocompromising diseases and cancers [22]. However, there is no direct guidance for administering filgrastim for patients suffering from CIA. Filgrastim/G-CSF is available in 300 mcg or 480 mcg vials or single dose syringes with recommended starting dose 5mcg/kg/day [22]. It is recommended to be administered up to 2 weeks or until ANC has reached 10,000/mm^3^ and is to be discontinued if ANC surpasses 10,000/mm^3^ after expected nadir [22].

In our patient, treatment of CIA took a total of 10 days of which Neupogen was dosed a total of 6 days with dosages of 300 mcg and 800 mcg (see Table 1) for patient recovery to a normal range of WBC 7 L and ANC 3.97 *μ*L. Further study may provide more efficient dosing and thus more expedient and cost-effective care.

There also continues to be nebulous guidelines in management of CIA through the utilization of bone marrow aspirate and biopsy. Current protocol in the initial studies for the diagnosis of CIA includes CBC, white cell differential, examination of the peripheral smear, observation of recovery after cessation of the drug in a healthy patient, bacterial and/or viral studies if the patient is febrile, and a bone marrow aspiration and biopsy [12, 14]. As the utilization of the bone marrow biopsy is to ascertain patient granulopoiesis status, physicians may choose to begin filgrastim/G-CSF treatment prior to performing a bone marrow aspirate. For our case, hematology recommended bone marrow biopsy on Treatment day 3 to rule out WBC aplasia; however, they suggested performing the biopsy only if the increased filgrastim dose to 800 mcg did not prove effective in improving neutrophils levels. When peripheral blood screening revealed cellular morphology categorized as normal, a bone marrow biopsy was not performed on our subject. Importantly, upon literature review, there are no stated guidelines on *when* to perform the bone biopsy [10, 14].

### 3.3. Valproic Acid and CYP Effects

Finally, while clozapine was immediately discontinued upon admission, valproate was continued throughout the duration of treatment for the patient's schizoaffective disorder (see Table 1). There has been suggestion that valproate may be a clozapine metabolism inducer [23], the serum valproate level was subtherapeutic 36.5 (50-100) on treatment day 0/admission to the medicine floor, and the clozapine level was not taken at admission; so, no definitive conclusion can be made. Other studies suggest that valproate is a weak inhibitor of CYP 3A4 [24, 25] and potent CYP 2C9 inhibitor [26, 27]. Additionally, in 2018, the Malik et al. case control study of 136 cases found an association of increased risk of CIA in those with concurrent use of sodium valproate and clozapine [28]. These findings might suggest that our subjects' concomitant dosing of valproate with her clozapine could have exacerbated the clozapine effect causing CIA. Should these theories prove true on larger study, the decision to continue the patient's prescription of valproate during treatment for CIA could have elongated the recovery process due to the CYP inhibitor effects of valproate on clozapine levels. Future investigation for drug interaction of valproate with clozapine together should be studied. Should valproate prove to be a CYP inhibitor, or exacerbate CIA, standardized guidelines should indicate valproate be discontinued in future findings of CIA, and alternative therapeutics should be explored. Additionally, adjunctive use of lithium with clozapine has been initially investigated to mitigate the level of neutropenia for those experiencing CIA [29]. Additional study into adjunct dosing of lithium should be investigated. Concurrent use of valproate with clozapine, adjunct dosing of lithium, and other patient risk factors may need to be considered while determining proper individualized treatment.

## 4. Conclusion

Through this case of CIA in a patient with longstanding schizoaffective disorder, CKD, and HLA-DQB1 and HLA-DQ2 positivity, the importance of implementing a standardized screen which accounts for the renal function and HLA status is imperative for safe practices in prescribing clozapine for psychiatric disorders and mitigating the risk for CIA. Additionally, proper standardized monitoring of renal status through the treatment process may also mitigate risk of potential kidney failure. Further study is necessary to distinguish valproate's role in agranulocytosis singularly or in concert with clozapine. More detailed standardized guidelines would be beneficial for treatment of CIA, should it occur. As clozapine is known as a drug of last resort to be administered when there is failure of more traditional, and risk averse therapeutics, it is difficult to deny this treatment option, as it ultimately implies that all other treatment options have been exhausted. Proper screening and established protocol and guidelines could increase the level of monitoring in patients with known risk factors as revealed by the official screen. This risk factor screen and subsequent monitoring could increase the cost-effectiveness of clozapine treatment and decrease the incidence and morbidity of this feared adverse event.

## Figures and Tables

**Table 1:** Treatment course lab table.

Treatment day	Treatment day 0	Treatment day 1	Treatment day 2	Treatment day 3	Treatment day 4	Treatment day 5	Treatment day 6	Treatment day 7	Treatment day 8	Treatment day 9	Treatment day 10	Treatment day 11	Treatment day 12
Lab value													
ANC level (*μ*L)	0.05	0	0.01	0.01	0.01	0.01	0	0.02	0.04	0.57	3.97	11.51	10.38
WBC (L)	N/A	0.4	0.4	0.3	0.2	0.3	0.2	0.3	0.4	1.8	7	18.7	N/A
Temperature max	98.8	98.6	100.7	103.1	101	98.5	99.3	97.3	98.1	98.6	97.8	97.7	98.1
BP		127/79	144/77	110/59	113/48	131/59	101/53	129/61	127/65	119/64	130/75	117/65	130/70
Hgb (G/DL)	10.4	9.9	11	10.5	9.8	8.9	8.5	8	8	8.6	9.6	9.3	8.4
Hematocrit (%)	33.8	31.8	36.4	33.6	31.6	29.3	27.2	26.3	26.1	28.1	32.2	30.6	28.6
H&H	10.4/33.8	9.9/31.8	11.0/36.4	10.5/33.6	9.8/31.6	8.9/29.3	8.5/27.2	8.0/26.3	8.0/26.1	8.6/28.1	9.6/32.2	9.3/30.6	8.4/28.6
BUN	N/A	N/A	38	36	46	55	77	70	57	43	33	29	25
CR	N/A	N/A	1.57	1.93	2.3	2.8	3.2	2.45	1.98	1.69	1.75	1.55	1.59
eGFR	N/A	N/A	33.61	26.48	21.63	17.24	14.78	20.11	25.71	30.87	29.65	34.11	33.12
Valproate level	36.5 L			50.9 L									
Amlodipine dose	10 mg	10 mg	10 mg	Not given	Not given	10 mg	10 mg	10 mg	Refused	10 mg	10 mg	10 mg	10 mg
Docusate dose	Refused	200 mg	200 mg	200 mg	200 mg	200 mg	200 mg	200 mg	200 mg	200 mg	200 mg	200 mg	200 mg
PEG dose	17 grams	17 grams											
Valproate dose	500 mg t.i.d	500 mg t.i.d	500 mg t.i.d	500 mg t.i.d	500 mg t.i.d	500 mg t.i.d	500 mg t.i.d	500 mg t.i.d	500 mg t.i.d	500 mg t.i.d	500 mg t.i.d	500 mg t.i.d	500 mg t.i.d
Filgrastim dose	N/A	300 mcg	300 mcg	800 mcg	800 mcg	800 mcg	N/A	N/A	800 mcg	N/A	N/A	N/A	N/A

## Data Availability

The subject data used to support the findings of this study are included in the Table within the article. Prior studies used to support the findings of this study are cited at relevant places within the text as references [1–29].

## Abbreviations

CIA: Clozapine-induced agranulocytosis
ANC: Absolute neutrophil count
DVT: Deep vein thrombosis
CKD: Chronic kidney disease
AKI: Acute kidney injury
CYP: Cytochrome P450
EMS: Emergency medical services
ED: Emergency department
HLA: Human leukocyte antigen
G-CSF: Granulocyte-colony stimulating factor.
